# Efficient detection of eyes on potato tubers using deep-learning for robotic high-throughput sampling

**DOI:** 10.3389/fpls.2024.1512632

**Published:** 2024-12-19

**Authors:** L. G. Divyanth, Salik Ram Khanal, Achyut Paudel, Chakradhar Mattupalli, Manoj Karkee

**Affiliations:** ^1^ Center for Precision and Automated Agricultural Systems, Department of Biological Systems Engineering, Washington State University, Prosser, WA, United States; ^2^ Department of Plant Pathology, Mount Vernon Northwestern Washington Research and Extension Center, Washington State University, Mount Vernon, WA, United States

**Keywords:** tissue sampling robot, machine vision, molecular diagnostics, potato pathogens, FTA card, YOLO

## Abstract

Molecular-based detection of pathogens from potato tubers hold promise, but the initial sample extraction process is labor-intensive. Developing a robotic tuber sampling system, equipped with a fast and precise machine vision technique to identify optimal sampling locations on a potato tuber, offers a viable solution. However, detecting sampling locations such as eyes and stolon scar is challenging due to variability in their appearance, size, and shape, along with soil adhering to the tubers. In this study, we addressed these challenges by evaluating various deep-learning-based object detectors, encompassing You Look Only Once (YOLO) variants of YOLOv5, YOLOv6, YOLOv7, YOLOv8, YOLOv9, YOLOv10, and YOLO11, for detecting eyes and stolon scars across a range of diverse potato cultivars. A robust image dataset obtained from tubers of five potato cultivars (three russet skinned, a red skinned, and a purple skinned) was developed as a benchmark for detection of these sampling locations. The mean average precision at an intersection over union threshold of 0.5 (mAP@0.5) ranged from 0.832 and 0.854 with YOLOv5n to 0.903 and 0.914 with YOLOv10l. Among all the tested models, YOLOv10m showed the optimal trade-off between detection accuracy (mAP@0.5 of 0.911) and inference time (92 ms), along with satisfactory generalization performance when cross-validated among the cultivars used in this study. The model benchmarking and inferences of this study provide insights for advancing the development of a robotic potato tuber sampling device.

## 1 Introduction

**Figure 1:**
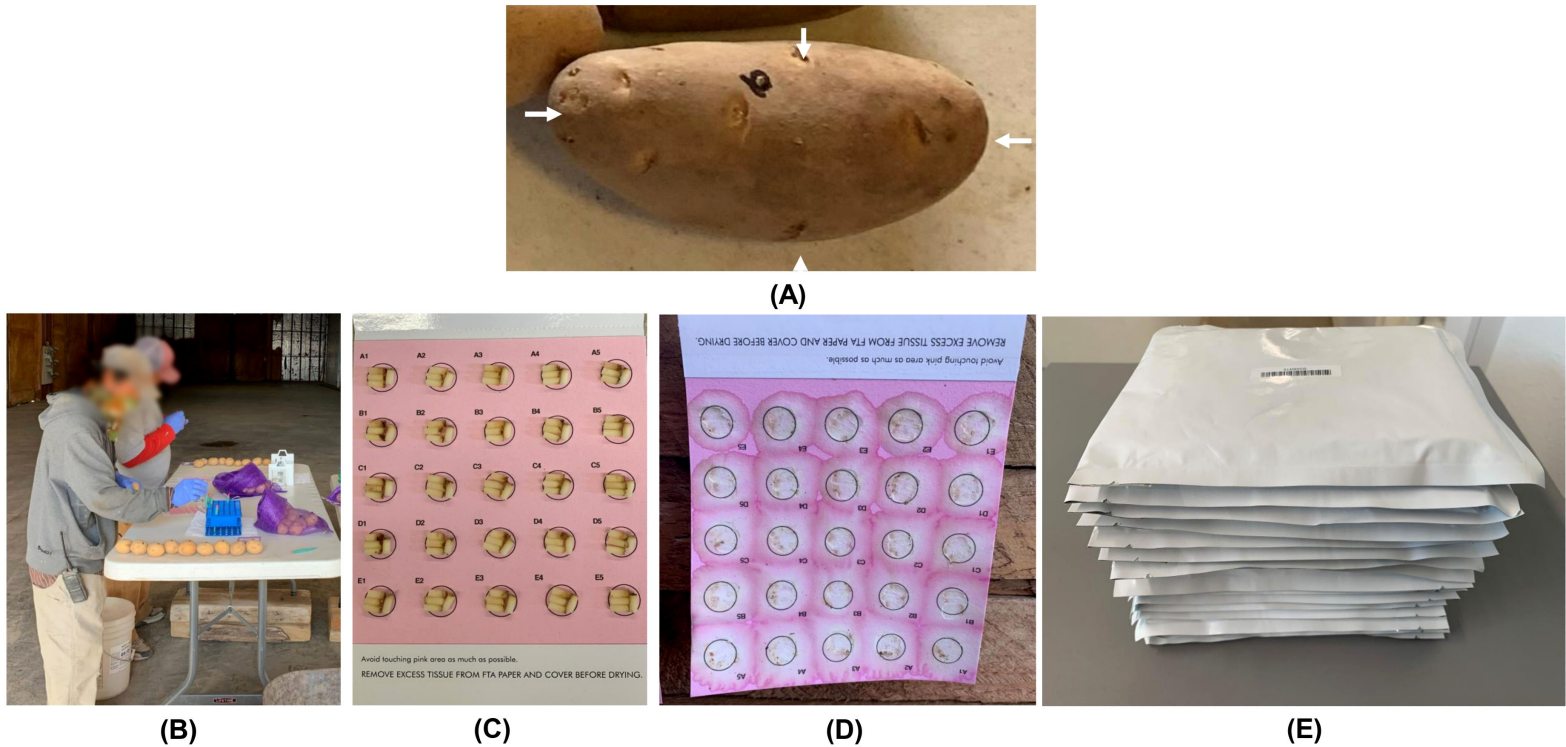
Potato tuber tissue sampling workflow **(A)** Desired sampling locations on a potato tuber indicated by four white arrows; **(B)** Manual sampling of potato tuber tissues onto FTA cards; **(C)** An FTA card containing samples from 25 potato tubers (four cores per tuber); **(D)** Nucleic acids from tuber tissue cores released onto the FTA card using a mechanical press; **(E)** FTA cards in envelopes ready to be shipped to a laboratory for downstream PCR-based pathogen detection.

**Figure 2:**
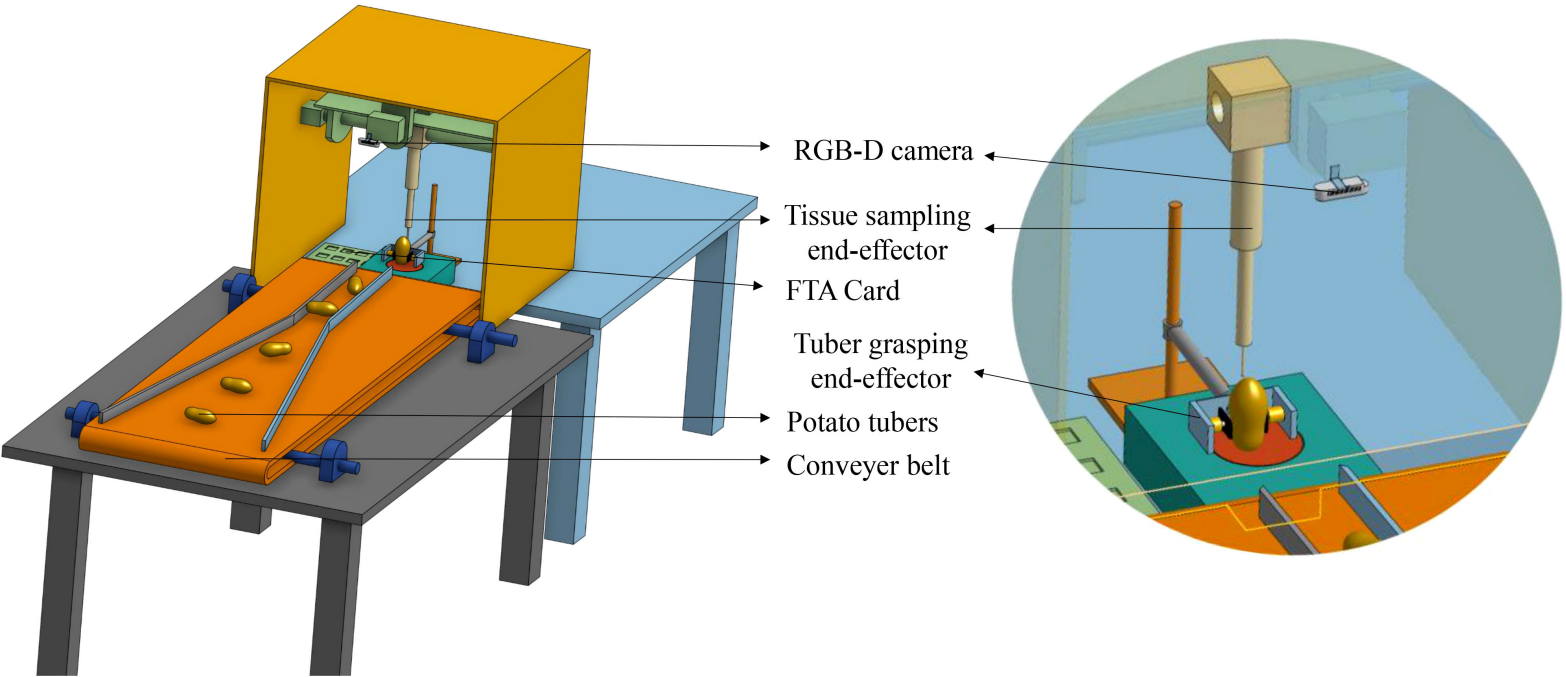
A prototype of the robotic tuber sampling platform. The camera captures tuber images that are used for detection of eyes and stolon scar on the tuber. The sampling tool performs sampling on the identified instances and places the tuber cores on the FTA card.

Potato is a high-value specialty vegetable grown in the United States and is worth about $5 billion per year (USDA National Agricultural Statistics Service, 2024). Potato is a vegetatively propagated crop where the risk of transmitting pathogens from one season to the next is typically greater compared to crops grown from true botanical seeds such as corn, soybean, or wheat. To maintain supply of healthy seed potatoes, there is a significant need to transition from traditional visual identification of diseases and serological methods of pathogen detection to a high throughput, sensitive, and specific molecular-based pathogen indexing approach (Mattupalli et al., 2022a; 2022b). Hence, a high-throughput workflow was developed, which involved sampling potato tubers onto Whatman FTA Plantsaver^®^ Cards (FTA), a format that eliminates the need for shipping bulky tubers to a laboratory. A crucial part of this workflow is the involvement of manual labor to sample four tuber tissue cores (one core from the terminal eye on the apical end of a tuber, and two cores arbitrarily from any two eyes on the longitudinal axis of a tuber, and a fourth core from the point of attachment of a tuber to the stolon) onto FTA cards that maximizes detection of potato pathogens such as potato virus Y (**Figure 1**). The samples on FTA cards are preserved at room temperature and then shipped to the laboratory for subsequent detection of pathogens using molecular approaches. However, this sampling process is physically strenuous, involving repetitive hand actions and requires about 8.5 person-hours to sample 400 tubers. As the potato industry moves towards adopting high-throughput molecular diagnostic approaches for detecting pathogens, there is an impending need to automate the tuber sampling process to save time and labor costs. However, physically demanding manual labor involved in the sampling process raises concerns about potential labor shortages and underscores the need for efficient and automated solutions. The integration of advanced computer vision and robotic technologies offers a compelling opportunity to automate the sample collection process. Such a prototype integrating a machine-vision subsystem for precise detection and localization of sampling locations on tubers, coupled with a manipulation subsystem and an end-effector for efficient sampling operations is shown in **Figure 2**.

The variability in the appearance of potato tubers and presence of soil adhering to tubers make it challenging to detect eyes and stolon scars. Conventional image processing-based object detection methods are very biased towards such factors (Ruiz et al., 2011; Wang et al., 2019; Wosner et al., 2021). Moreover, a robust model accounting for variations in color, texture, and morphological attributes is crucial for effectively detecting desired sampling locations across diverse potato cultivars. In recent years, deep-learning-based convolutional neural networks (CNNs) have made remarkable progress in object detection (Jiao et al., 2019; Sharma and Mir, 2020; Bhatti et al., 2022; Nawaz et al., 2022; Zaidi et al., 2022; Xiao et al., 2023; Nain et al., 2024). Due to their ability to be trained end-to-end without the need for explicit feature extraction, CNNs are better equipped to identify specific patterns in the image that are crucial for accurate eye and stolon scar detection. Deep learning object detectors fall into two categories: a) two-stage detectors, involving a preprocessing step for object proposals followed by classification; and b) single-stage detectors that are end-to-end without region proposals (Sharma and Mir, 2020; Soviany and Ionescu, 2018).

Single-stage detectors, such as You Only Look Once (YOLO), are known for their computational efficiency, faster inference, and suitability for real-time applications, especially on resource-constrained embedded devices (Carranza-García et al., 2020; Dang et al., 2023; Verma et al., 2021). Originally proposed by Redmon et al. (2015), YOLO has evolved through several versions, including YOLO9000, YOLOv3, YOLOv4, Scaled-YOLOv4, YOLOv5, YOLOR, YOLOv6, YOLOv7, YOLOv8, YOLOv9, YOLOv10, and YOLO11. Each iteration introduces architectural improvements to tackle specific challenges in object detection (Jiang et al., 2022; Terven et al., 2023; Morbekar et al., 2020). While several studies have employed YOLO-based models for agricultural tasks like fruit detection (Rathore et al., 2023), weed identification (Dang et al., 2023), and disease detection (Morbekar et al., 2020), limited research has been dedicated to the precise identification of small, intricate features such as eyes and stolon scars on potato tubers. These features are crucial for targeted, robotic sampling of tubers but present unique challenges due to their variability in size, shape, and appearance, particularly across different cultivars.

Therefore, this research focused on addressing the lack of effective, high-throughput methods for detecting these specific features, which has not been thoroughly explored in the context of automated sampling for pathogen detection. This study evaluates and benchmarks a range of YOLO-based object detectors (from YOLOv5 to YOLO11) specifically for this purpose, testing their accuracy, speed, and robustness on five potato cultivars. By identifying the most suitable model, our findings provide a foundation for its integration into high-throughput robotic tuber sampling frameworks, thereby enhancing the precision and efficiency of the tuber sampling process. The primary contributions of this research are:

- A comprehensive comparison of YOLO-based object detectors to assess their effectiveness at detection sampling locations (eyes and stolon scars) on potato tubers, focusing on detection speed, accuracy, and robustness.
- Creation of a robust image dataset comprising tubers from five potato cultivars with different skin colors, which serves as a benchmark for the development of a robotic tuber sampling system.

## 2 Materials and methods

### 2. 1 Potato tuber images dataset

**Figure 3:**
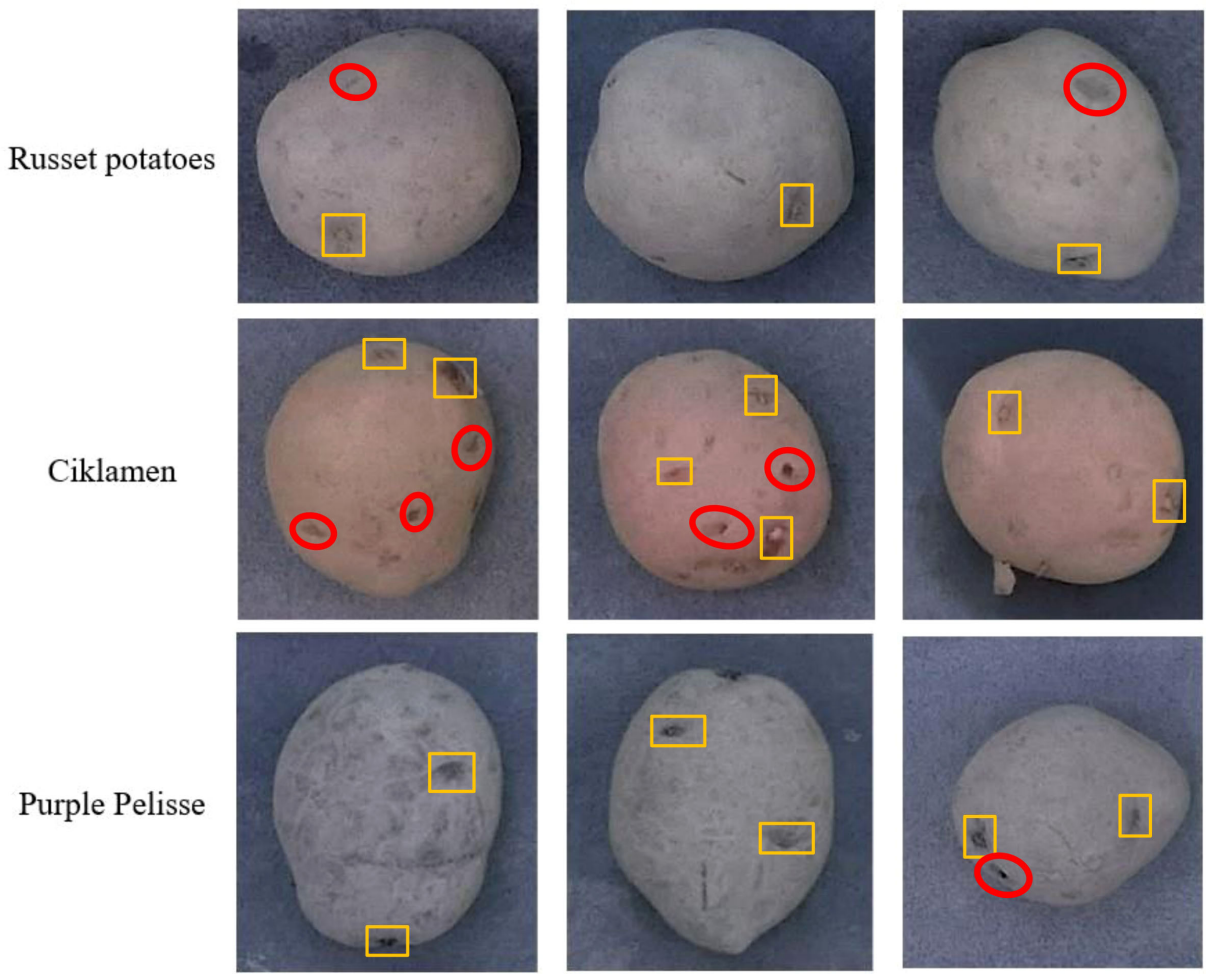
Example images of potato tubers used to develop models for the detection of eyes and stolon scar. The yellow polygons denote the bounding boxes of the eyes. Red ovals denote regions that exhibit attributes similar to the eyes.

**Figure 4:**
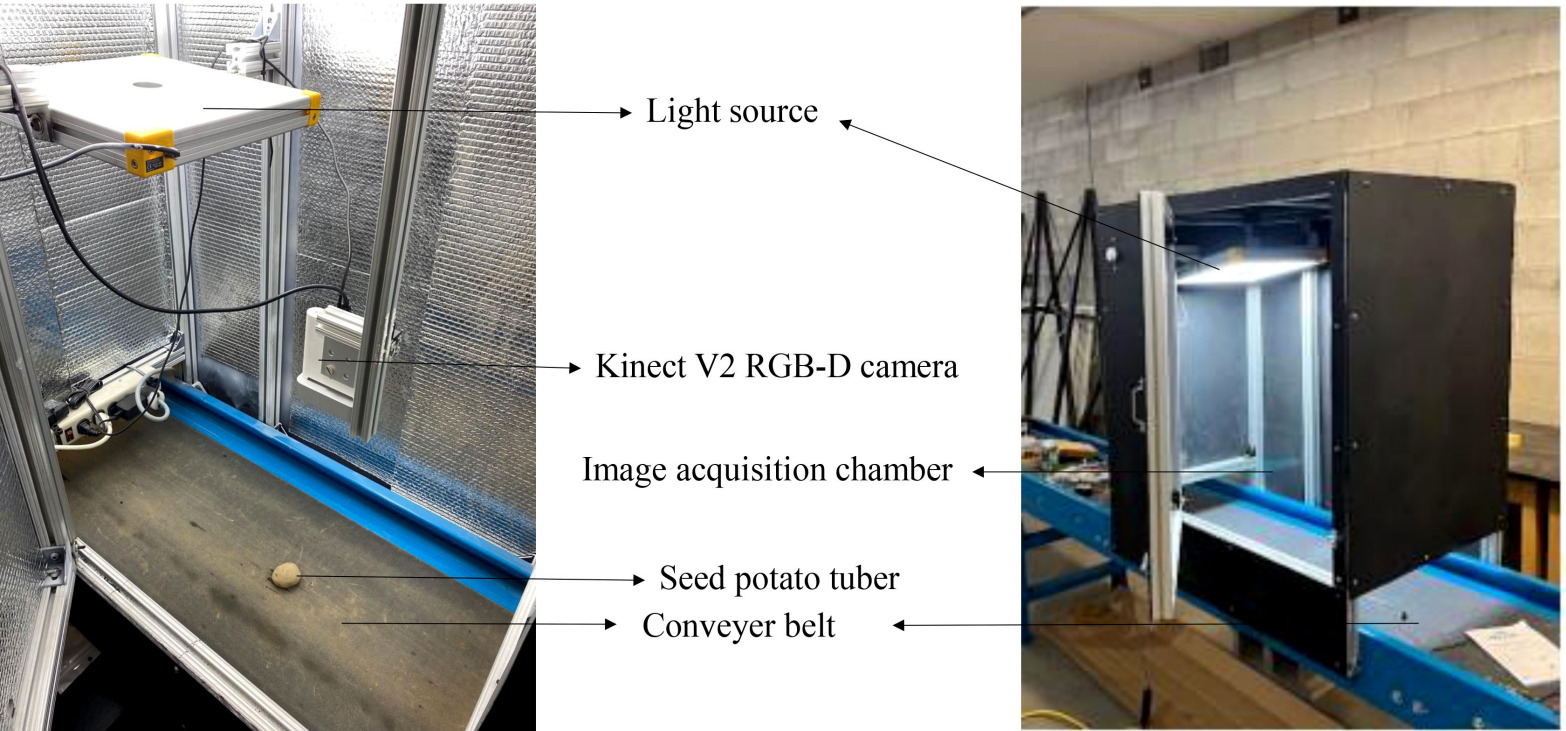
The image acquisition setup used for collecting potato tuber images dataset.

**Figure 5:**
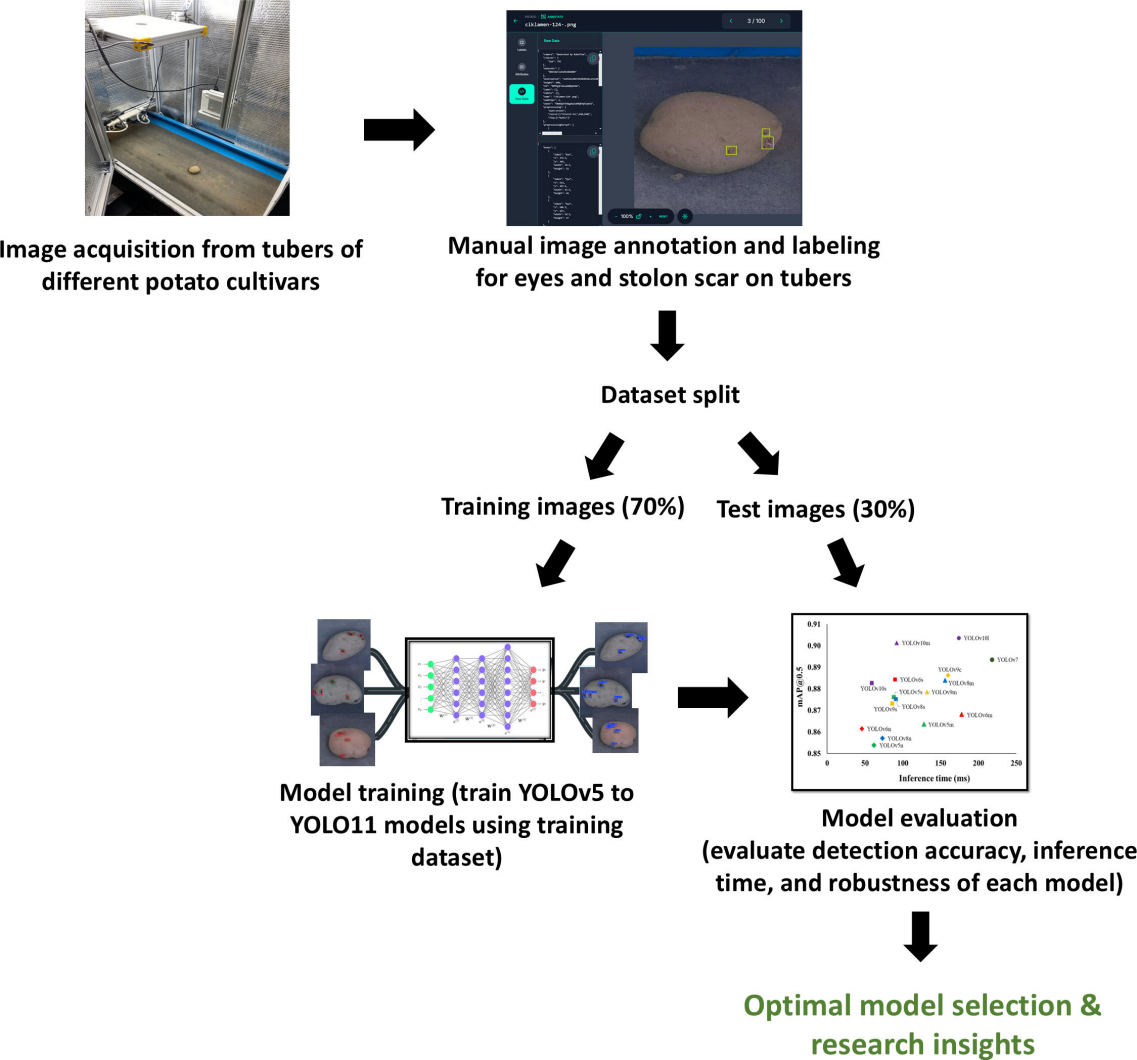
Workflow of the study outlining the development and evaluation process of identifying optimal object detection models for detecting eyes and stolon scars on potato tubers.

The dataset comprised images of different potato cultivars, including Umatilla Russet, Russet Burbank, and Ute Russet (russet skin), Ciklamen (red skin), and Purple Pelisse (purple skin) (**Figure 3**). Image acquisition was conducted in a controlled indoor setup using a conveyor belt system with an imaging chamber (0.75 m × 0.5 m × 0.6 m H×W×). The chamber was constructed from aluminum profiles, with reflective aluminum lining along the inner walls to enhance light uniformity. The image acquisition setup features an accessible design, allowing easy adjustments to the camera’s height relative to the conveyor belt for optimal image capture. To ensure consistent lighting conditions, a 5000-6500K white light source was employed, complemented by the reflective properties of the inner chamber walls to evenly distribute light across the tuber surface. This setup ensured uniform illumination, minimizing shadows and glare. The image acquisition utilized only the RGB channels from the Microsoft Kinect V2 RGB-D camera sensor (Microsoft Inc., Redmond, WA, USA), as shown in **Figure 4**. The images were collected by flipping the tubers to expose the alternate sides, ensuring a comprehensive dataset capturing the variations in eye distribution and appearance across the entire potato surface. The workflow of this study is illustrated in **Figure 5**.

A total of 300 tuber images were collected for each skin color, resulting in a dataset comprising 900 images. Among these, 70% of the images from each tuber skin color were randomly allocated for training the models, while the remaining 30% of the images were reserved as test set. The images were then annotated for the eyes and stolon scar using the hasty.ai software (Hasty Inc., Berlin, Germany). This interface provided a wide range of advanced annotation tools such as the object detection assistance that facilitated the creation of ground truths quickly. Sample images from the dataset are provided in **Figure 3**. Furthermore, data augmentations based on image geometry, including flipping about x- and y-axis, translation ( ± 25%), scaling ( ± 25%), mosaic, and rotations ( ± 30°), as well as image intensity such as brightness variation (with coefficients of 0.7, 0.8, 0.9, 1.1, 1.2, and 1.3), HSV variation (with fractions of H = 0.015; S = 0.7; V = 0.4), and Gaussian blurring (with a standard deviation of 1.5) were performed on the images in the training set, while preserving and replicating the bounding box (ground-truth) information in the augmented images. These augmentations were carefully chosen to reflect real-world conditions that the tuber sampling robot will potentially encounter, enhancing detection accuracy and resilience across various field conditions. For example, brightness and HSV variations enhance model robustness to different lighting environments, while Gaussian blurring prepares the model to handle potential motion blur from the vision system mounted on a moving sampling end-effector.

### 2.2 Deep-learning-based object detectors

Deep-learning object detectors typically consist of two key network components: a backbone responsible for extracting image features from high-dimensional input, and a head component dedicated to regressing the coordinates of identified objects’ bounding boxes and predicting their respective classes (Sharma and Mir, 2020). The YOLO object detectors employ a single-stage head, performing simultaneous classification and localization of semantic objects through dense sampling (Redmon et al., 2015). The neck part of the network, which includes some intermediary layers positioned between the backbone and the head, is designed to gather feature maps from different stages. The advancements in the YOLO family object detectors primarily revolve around enhancing the backbones, refining feature integration models, and optimizing the network training techniques, including the hyper-parameters (Diwan et al., 2023).

YOLOv3 aimed to strike a balance between speed and accuracy for real-time object detection, surpassing its predecessors (Redmon and Farhadi, 2017, 2018). It utilized the Darknet-53 network as its backbone for efficient feature extraction from input images. Notably, YOLOv3 included a simplified variant, YOLOv3-tiny, whose backbone was based on the Darknet-19 architecture. The YOLOv4 model (Bochkovskiy et al., 2020) was introduced in 2020, bringing significant advancements to the YOLO framework. This version featured the CSPNet (Wang et al., 2020) backbone and introduced spatial pyramid pooling (SPP) (He et al., 2014) and path aggregation network (PAN) block (Liu et al., 2018) necks. YOLOv4 aimed to enhance detection accuracy through innovative training methods, including the bag of freebies such as CutMix (Yun et al., 2019) and DropBlock regularization (Ghiasi et al., 2018), and bag of specials like mish activation and distance-IoU-based non-maximum suppression (DIoU-NMS) (Zheng et al., 2020). Notably, YOLOv4-large scaled for cloud GPUs achieved state-of-the-art detection accuracy on the COCO dataset (Lin et al., 2014). In 2021, Wang et al. (2021) presented the Scaled-YOLOv4, which extended the capabilities of YOLOv4 for deployment on various computing devices. The model was based on the cross-stage partial network for effective model scaling. YOLOR (Wang et al., 2021) integrated implicit and explicit knowledge to learn a general, unified representation for multiple tasks, such as joint object detection and multi-label image classification. It achieved comparable accuracy to Scaled-YOLOv4 but demonstrated a significant increase in inference speed.

Further, YOLOv5 was released by Ultralytics LLC, that claimed superiority over its predecessors in performance. A notable modification in YOLOv5 was the integration of the anchor box selection process. YOLOv6 (Li et al., 2022) was released in 2022, which featured a set of renovated designs, especially in network architecture, label assignment, and loss functions. These changes aimed to optimize the model for industrial deployment, reflecting the ongoing evolution of the YOLO framework. Then, YOLOv7 (Wang et al., 2023) implemented an extended efficient layer aggregation network, utilizing various trainable bag-of-freebies methods, including planned re-parameterization and coarse-to-fine lead guided label assignment. YOLOv7 notably surpassed the performance of previous object detectors in both speed and accuracy on the COCO dataset. Further, YOLOv8 was proposed by the Ultralytics community and validated for superior accuracy on Microsoft COCO and Roboflow 100 datasets. YOLOv8’s developer-friendly attributes, such as an intuitive Command Line Interface (CLI) and a well-organized Python package enhance the overall ease of implementation (Khare et al., 2023; Reis et al., 2023). YOLOv9 introduced several model variants, including YOLOv9n, YOLOv9s, YOLOv9m, YOLOv9c, and YOLOv9x, achieving mean average precision (mAP) scores from 39.5% to 54.4% on the MS-COCO dataset (Wang et al., 2024b). Building on these improvements, YOLOv10 included variants like YOLOv10n, YOLOv10s, YOLOv10m, YOLOv10b, YOLOv10l, and YOLOv10x (Wang et al., 2024a). It achieved mAP scores ranging from 38.5% to 54.4%, with significant reductions in latency. YOLO11, the latest iteration in the YOLO series of object detection models, builds upon the advancements of YOLOv8 by introducing key architectural innovations and parameter optimizations, such as the C3k2 (Cross Stage Partial with kernel size 2) block, SPPF (Spatial Pyramid Pooling - Fast), and C2PSA (Convolutional block with Parallel Spatial Attention) (Jocher and Qiu, 2024). These components significantly improve feature extraction, contributing to faster and more accurate detection performance.

In this study, we selected seven state-of-the-art YOLO object detectors described above, namely, YOLOv5, YOLOv6, YOLOv7, YOLOv8, YOLOv9, YOLOv10, YOLO11, and their variants to develop models for detection of eyes on potato tubers. To optimize deployment in real-time, YOLO variants with less than 40 million parameters were chosen, prioritizing models that provide a balance between computational efficiency and accuracy. The YOLO detectors selected for this study utilized open-source software packages developed by their creators.

### 2.3 Modelling and experimentation

The original image annotations which were in JavaScript Object Notation (JSON) format were imported to the Roboflow software (Roboflow Inc., Des Moines, IA, USA) and converted to YOLO format labels. The models were trained via transfer learning by fine-tuning the weights learnt using the COCO dataset (Lin et al., 2014). The input size of the image was set to 640 × 640 pixels, as dictated by the YOLO architectures. The models were trained for 100 epochs with a mini-batch size of 16 images using the PyTorch framework. The learning rate of the networks was adjusted using the cosine annealing method, whereas the other hyperparameter options used the default values of the respective models’ official implementations. The models were trained and evaluated using Alienware 15 R3 system with Nvidia GeForce GTX 1060 8GB GPU on an Ubuntu operating system. Furthermore, the models were evaluated for their overall performance and cultivar-wise cross-validation using the image datasets obtained from different cultivars.

### 2.4 Performance evaluation metrics

The performance of the YOLO models for detection of tuber eyes was assessed based on their detection accuracy, complexity of the model, computational cost, and inference time, as described below.

The performance of the models for detecting eyes on tubers was evaluated using metrics including precision (P), recall (R), and mean average precision (mAP, which includes mAP@0.5 and mAP@0.5:0.95). If the true positives, false positives, true negatives, and false negatives are represented by TP, FP, TN, and FN, respectively, then P and R were defined as (Equations 1, 2):


(1) $$P=\;\frac{TP}{TP+FP}$$

(2) $$R=\;\frac{TP}{TP+FN}$$

The AP is defined as the area under the precision-recall (P-R) curve, generated by plotting precision (P) on the vertical axis and recall (R) on the horizontal axis (Equation 3). AP serves as a comprehensive metric, determining the overall efficacy of the model to detect eyes on potato tubers. Intuitively, mean average precision (mAP) is calculated as the mean of AP values for each class objects in the dataset. Given that the detection model in this study is designed for single-class object detection, the mAP is simply the AP of that specific class. The mAP@0.5 represents the mAP value at an intersection over union (IoU) threshold of 0.5, whereas the mAP@0.5:0.95 represents the average mAP over different IoU thresholds, from 0.5 to 0.95 with steps of 0.05.


(3) $$AP_{c}=\;\int_{0}^{1}P\left(R_{c}\right)dR_{c}$$

The complexity can be measured as the number of parameters in the model, which plays a crucial role for real-time deployment. Larger models with a high number of parameters demand more memory space and resources. The number of parameters also directly impacts the memory requirements, computation costs, and inference times. The inference time, representing the duration for a trained model to make predictions on an input image, is one of the key considerations for real-time deployment. The inference time for each YOLO detector was determined by averaging the time required to predict all images within the test set.

## 3 Results

### 3.1 Performance of YOLO models

Based on the cut-off set for the number of parameters in the network (i.e., 40 million), nineteen different YOLO architectures [YOLOv5n, YOLOv5s, YOLOv5m, YOLOv6n, YOLOv6s, YOLOv6m, YOLOv7 (base model), YOLOv8n, YOLOv8s, YOLOv8m, YOLOv9s, YOLOv9m, YOLOv9c, YOLOv10s, YOLOv10m, YOLOv10l, YOLO11s, YOLO11m, YOLO11l (where n = nano, s = small, m = medium, c = compact, and l = large)] were evaluated for their performance detecting eyes and stolon scars. The models exhibited encouraging training performance with convergence at about 0.80–0.90 mAP@0.5 achieved within 50 epochs. However, there was no significant improvement in the accuracies after this point, confirming that training for 100 epochs is sufficient for this study. The eye detection performance of all the models is presented in **Table 1**. The average results from three independent runs of each model were recorded. In general, all the nineteen models showed appreciable accuracies with mAP@0.5 in the range of 0.854–0.914 and precision values in the range of 0.832–0.903. The best and the least performing models in terms of precision, recall, mAP@0.5, and mAP@0.5:0.95 were the YOLOv10l and YOLOv5n respectively. In general, the performance of different YOLO architectures followed the order: YOLOv10 > YOLOv7 > YOLOv9 > YOLOv6 > YOLO11 > YOLOv8 > YOLOv5, however, the differences in performance were marginal. The YOLOv10 models, particularly YOLOv10l, achieved the highest precision at 0.903, indicating that they are the most precise at identifying eyes and stolon scar. This was closely followed by YOLOv10m and YOLOv7, with precision values of 0.901 and 0.883, respectively. In contrast, the YOLOv5 and YOLOv8 models generally have lower precision, with YOLOv5n and YOLOv8n at around 0.832. On the other hand, the highest recall was observed with YOLOv9c at 0.821, suggesting it is the most effective at identifying all relevant instances. YOLOv7 and YOLOv10 models also show strong recall, with YOLOv7 achieving 0.817 and YOLOv10l at 0.815.

**Table 1:** Potato tuber eyes and stolon scar detection performance of the YOLO object detectors on the test dataset.

YOLO model	Precision	Recall	mAP@0.5	mAP@0.5:0.95
YOLOv5n	0.832	0.752	0.854	0.495
YOLOv5s	0.862	0.788	0.876	0.529
YOLOv5m	0.839	0.754	0.864	0.501
YOLOv6n	0.845	0.769	0.862	0.512
YOLOv6s	0.875	0.811	0.884	0.531
YOLOv6m	0.841	0.772	0.868	0.510
YOLOv7	0.883	0.817	0.894	0.536
YOLOv8n	0.832	0.759	0.857	0.489
YOLOv8s	0.852	0.784	0.875	0.525
YOLOv8m	0.858	0.787	0.884	0.527
YOLOv9s	0.849	0.774	0.873	0.516
YOLOv9m	0.853	0.774	0.878	0.529
YOLOv9c	0.875	0.821	0.886	0.533
YOLOv10s	0.862	0.807	0.883	0.523
YOLOv10m	0.901	0.810	0.911	0.547
YOLOv10l	0.903	0.815	0.914	0.549
YOLO11s	0.860	0.791	0.877	0.524
YOLO11m	0.867	0.785	0.882	0.529
YOLO11l	0.871	0.798	0.884	0.532

**Figure 6:**
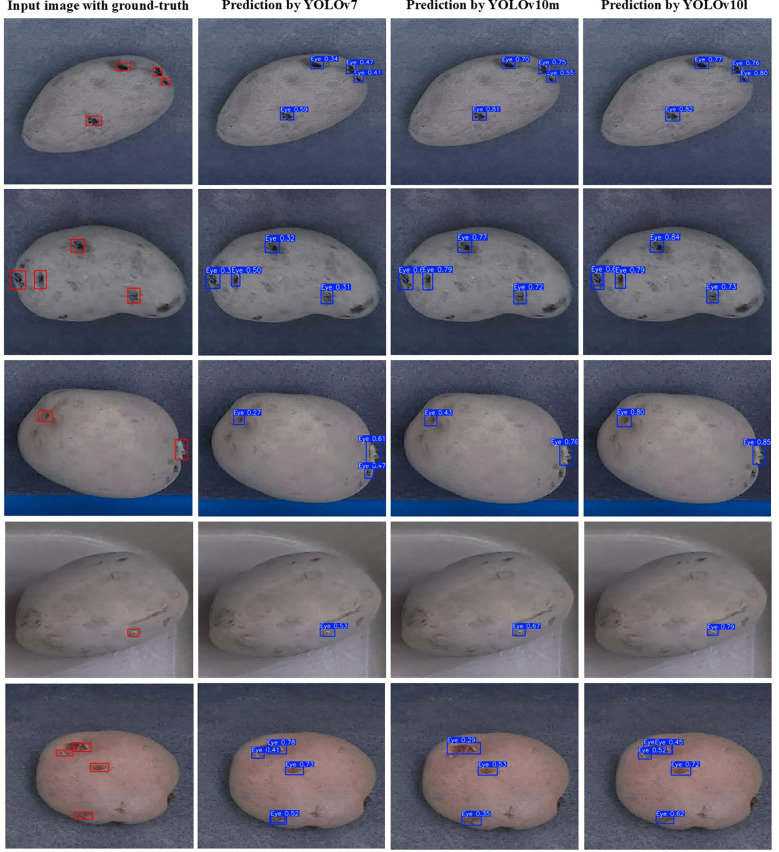
Examples of potato tuber images with the ground-truth (red boxes) and predicted bounding boxes (blue boxes) of the eyes and stolon scar.

Seventeen out of the nineteen models produced mAP@0.5:0.95 greater than 50%, while YOLOv10m and YOLOv10l recorded mAP@0.5 over 90%. YOLOv6s, YOLOv7, YOLOv9c, and YOLO11l demonstrated similar overall performance to YOLOv10m and YOLOv10l models in terms of precision, recall, and mAP while exhibiting smaller gaps in mAP@0.5 and mAP@0.5:0.95, which suggested that these models achieved better generalization to different IoU thresholds. Some example images with the predictions of the best performing models, i.e., YOLOv7, YOLOv10m, and YOLOv10l, are shown in **Figure 6**. The predictions yielded by these models were visually appreciable for russets, red, and purple skinned cultivars, demonstrating the efficacy of the selected object detectors for eyes and stolon scar detection on potato tubers. The confidence scores of the YOLOv10l model were higher than those of YOLOv10m, which in turn outperformed YOLOv7, reflecting a direct correlation of the trend with the models’ respective precision values. This trend highlights the improved performance of larger models to accurately identify objects and suggests that model complexity plays a key role in enhancing detection reliability.

YOLOv10’s lightweight classification heads are designed to maintain efficiency without sacrificing accuracy. The YOLOv10m model, with significantly fewer parameters (15.4 million) compared to other medium-scale models like YOLOv5m (21.2 million), YOLOv6m (34.9 million), YOLOv8m (25.9 million), and YOLOv9m (20 million), strikes a balance between model complexity and task-specific requirements. This streamlined architecture allows YOLOv10m (mAP@0.5 = 0.911) to outperform its smaller counterpart, YOLOv10s (mAP@0.5 = 0.883) at detecting eyes and stolon scar on the tubers. Thus, in the YOLOv10 series, the medium-scale model effectively leverages its computational power to enhance performance, demonstrating a clear advantage over the small-scale model. Moreover, the performance difference between YOLOv10m and YOLOv10l was marginal, with YOLOv10l performing only slightly better.

### 3.2 Potato cultivar-specific cross-validation performance of the YOLO models

Different potato cultivars can exhibit variations in size, shape, and skin color posing challenges for object detection models. Cross validation among distinct cultivars serves to assess a model’s capacity to generalize to new types of tubers not encountered during training. During cross-validation, all images of one cultivar were used as the test set while tuber images of the other two cultivars were used for training. **Table 2** presents the validation results of the top-performing models in terms of mAP@0.5 score. The cross-validation performances were lower compared to when images from every cultivar were included in the dataset (as shown in the last column of **Table 2**). Particularly, the models exhibited the poorest performance identifying eyes and stolon scar on Purple Pelisse cultivar tubers. The comparable mAP observed during cross-validation for russet and red skin cultivars suggests that the features learned from one can effectively aid in the recognition of eyes in the other. This finding also indicates that the models have learned a robust feature representation that is transferable between russet skin and red skin potato cultivars. YOLOv7 achieved the highest mAP@0.5 of 0.781 for russets, 0.809 for ‘Ciklamen’, and 0.742 for ‘Purple Pelisse’. Among the YOLOv10 models, YOLOv10m achieved an mAP@0.5 of 0.762 for russet, 0.783 for ‘Ciklamen’, and 0.701 for ‘Purple Pelisse’. The YOLOv10l variant showed slight improvements, with an mAP@0.5 of 0.772 for russet, 0.796 for ‘Ciklamen’, and 0.723 for ‘Purple Pelisse’. These results showed the effectiveness of the YOLOv10 architecture, especially to accurately distinguish subtle features. In contrast, the YOLOv5 and YOLOv8 models exhibited relatively lower generalizability, as indicated by their reduced performance during cross-validation.

**Table 2:** Cultivar-wise cross-validation results (mAP@0.5) of YOLO detectors for detection of eyes on potato tubers.

YOLO model	Russets	Ciklamen	Purple Pelisse	Overall
YOLOv5s	0.735	0.721	0.651	0.876
YOLOv5m	0.740	0.759	0.650	0.864
YOLOv6s	0.768	0.798	0.709	0.884
YOLOv6m	0.773	0.796	0.717	0.868
YOLOv7	0.781	0.809	0.742	0.894
YOLOv8s	0.749	0.720	0.643	0.875
YOLOv8m	0.759	0.751	0.642	0.884
YOLOv9m	0.752	0.768	0.675	0.878
YOLOv9c	0.764	0.780	0.690	0.886
YOLOv10m	0.762	0.783	0.701	0.901
YOLOv10l	0.772	0.796	0.723	0.904
YOLO11s	0.741	0.735	0.624	0.877
YOLO11m	0.759	0.744	0.653	0.882
YOLO11l	0.760	0.763	0.668	0.884

The images of the specified cultivar were used to test the model that was trained using images of the other two cultivars. The overall mAP@0.5 are the same values as in **Table 1**, provided here for comparison with the cross-validation results.

**Figure 7:**
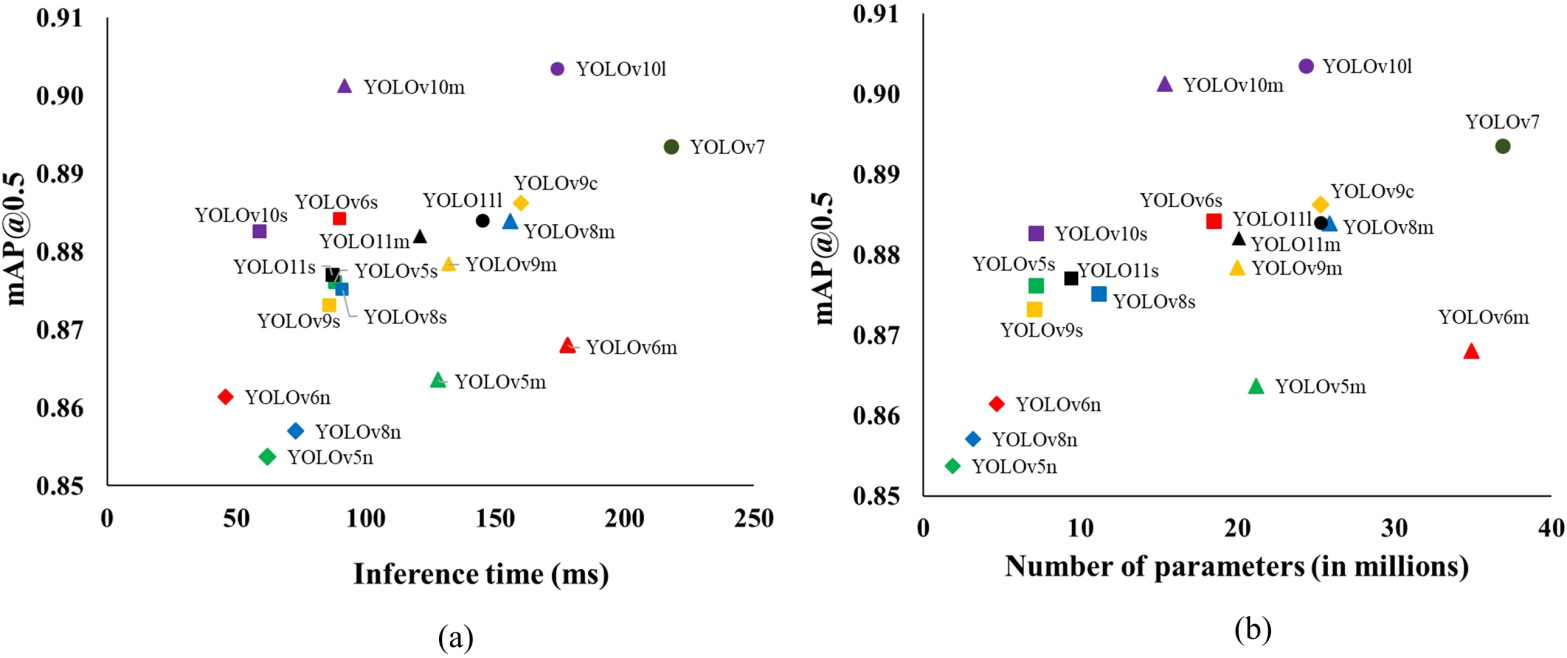
The inference time (ms) **(A)** and number of parameters (millions) **(B)** versus mAP@0.5 for different YOLO object detectors for detecting eyes on potato tubers. The models belonging to the same YOLO detector are labeled with the same marker shapes and color.

Balancing the models’ detection capability, complexity, and inference time is crucial for achieving real-time performance during practical deployment. The subplots in **Figure 7** depict the trade-offs between these factors by plotting inference time and number of parameters against detection performance measured in terms of mAP@0.5. While these inferences were performed on high-performing hardware, a similar trend can be expected in embedded systems. Notably, there is a linear positive relationship between the inference time and the number of parameters in the models, a trend consistent with findings from previous studies (Junos et al., 2021; Mirhaji et al., 2021; Kohli et al., 2022; Dang et al., 2023). Among the nineteen models, YOLOv7 had the highest inference time of about 218ms, followed by YOLOv6m (178ms) and YOLOv10l (174ms). YOLOv10m stood out with a strong balance of high accuracy (mAP@0.5 = 0.901) and a lower inference time of 92ms, making it the model with the lowest latency among those with high accuracy.

## 4 Discussion

The assessment of nineteen different versions of the YOLO architectures (YOLOv5, YOLOv6, YOLOv7, YOLOv8, YOLOv9, YOLOv10, YOLO11) for combined eye and stolon scar detection from potato tubers provided crucial insights pivotal for advancing the development of a robotic tuber sampling device. Subsequent evaluation in terms of precision, recall, and mAP@0.5 demonstrated that YOLOv10 models outperformed other architectures, closely followed by YOLOv7 and YOLOv9. Notably, YOLOv10m stood out with significantly fewer parameters and lower inference time as it was also comparable in terms of detection results. The nano-scale models of the YOLO versions (YOLOv5n, YOLOv6n, YOLOv8n) showed the least performance as compared to small- and medium-scale networks (**Table 1**). This suggests that these models are too small to capture the complex attributes of tuber eyes and stolon scar and differentiate them from other similar structures such as mud, soil aggregates, and surface inequalities present on the tuber surface, leading to diminished performance and generalization capability. Furthermore, they are more prone to overfitting and capturing noise in the training data rather than generalizing to new unseen potato tubers.

It is interesting to note that in some YOLO architectures, such as YOLOv5 and YOLOv6, the medium-scale models did not outperform their small-scale counterparts. For instance, YOLOv6s achieved a higher mAP@0.5 of 0.884, compared to YOLOv6m’s mAP@0.5 of 0.868. Similarly, within the YOLOv5 series, the small-scale model YOLOv5s outperformed the medium-scale YOLOv5m, with mAP@0.5 of 0.876 versus 0.864, respectively. For other architectures like YOLOv8, YOLOv9, and YOLO11, the performance improvement from small-scale to medium-scale models was negligible. The increase in mAP@0.5 was only 1.02% for YOLOv8 (from YOLOv8s to YOLOv8m), 0.57% for YOLOv9 and YOLO11 (from YOLOv9s to YOLOv9m and YOLO11s to YOLO11m). These marginal improvements contrast with results observed by other researchers using benchmark datasets like COCO, as well as findings from other studies where medium-scale models generally outperformed small-scale models (Dang et al., 2023; Reis et al., 2023; Wang et al., 2024a, 2024). These observations suggest that the specific dataset and task being addressed in this study may not fully utilize the additional capacity of medium-scale models. The size and complexity of the dataset might not be sufficient to leverage the increased model capacity, leading to a scenario where smaller models, which are more efficient and faster, perform equally well or even better to detect eyes and stolon scar on potato tubers.

**Figure 7A** depicts there is a considerable trade-off between the mAP@0.5 and inference time that needs to be considered during model selection. For instance, YOLOv7, despite having the highest mAP@0.5 of 0.894, also has the longest inference time at 218ms. Similarly, models like YOLOv10l and YOLOv9c, which achieve high mAP@0.5 scores of 0.904 and 0.886, respectively, also come with comparatively higher inference times (174ms for YOLOv10l and 160ms for YOLOv9c). On the other hand, the nano-versions of the YOLO detectors, such as YOLOv6n and YOLOv8n, are the most computationally efficient, with inference times under 80ms. However, this efficiency comes at the expense of detection performance. Interestingly, models with a moderate number of parameters, such as YOLOv6s and YOLOv10m, offer a promising balance (**Figure 7B**). YOLOv6s achieves an impressive mAP@0.5 of 0.884 with an inference time of just 90ms, closely matching the accuracy of YOLOv7 but with significantly fewer parameters and a lower inference time. Similarly, YOLOv10m, with an mAP@0.5 of 0.901, provides high accuracy while requiring almost half the inference time compared to its larger counterpart, YOLOv10l. Henceforth, models like YOLOv6s and YOLOv10m offer a balanced solution for deployment in the robotic sampling device with high accuracy and moderate inference times.

The cultivar-specific cross validation revealed notable disparities in the model’s ability to accurately detect eyes and stolon scar across different potato cultivars. Among the evaluated models, the YOLOv7, YOLOv10, and YOLOv6 variants emerged as the most robust in terms of generalizability across different tuber cultivars for eye and stolon scar recognition. Models developed based on images obtained from russet and red potato cultivars showed poor performance when tested on tuber images of Purple Pelisse cultivar. We hypothesize this effect to be due to the appearance of eyes in hues of purple or violet, which blended more with the surrounding tuber skin color. Another significant drawback of the current YOLO models is the combined detection of the eye and stolon scar as a single class. Identifying the stolon scar accurately is crucial, as it provides essential tissue and biological information necessary for high-throughput diagnosis. When both features are treated as a single class, there’s a high likelihood of missing the stolon scar during robotic sampling as the system would just sample four locations among all the detections, resulting in suboptimal sampling decisions. The models’ performance in multi-class detection has been notably poor due to the similar visual characteristics of the eye and stolon scar. This underscores the need for a more sophisticated dataset and training and inference strategies that enables the vision system to recognize these features as distinct entities. This involves enhancing image diversity by including more images of tubers oriented to clearly reveal the stolon scar on the surface. Additionally, incorporating a broader variety of potato cultivars will enable the model to learn the distinct features associated with each class more effectively. This approach will improve the model’s ability to differentiate between the eye and stolon scar, leading to more accurate detection outcomes.

**Figure 8:**
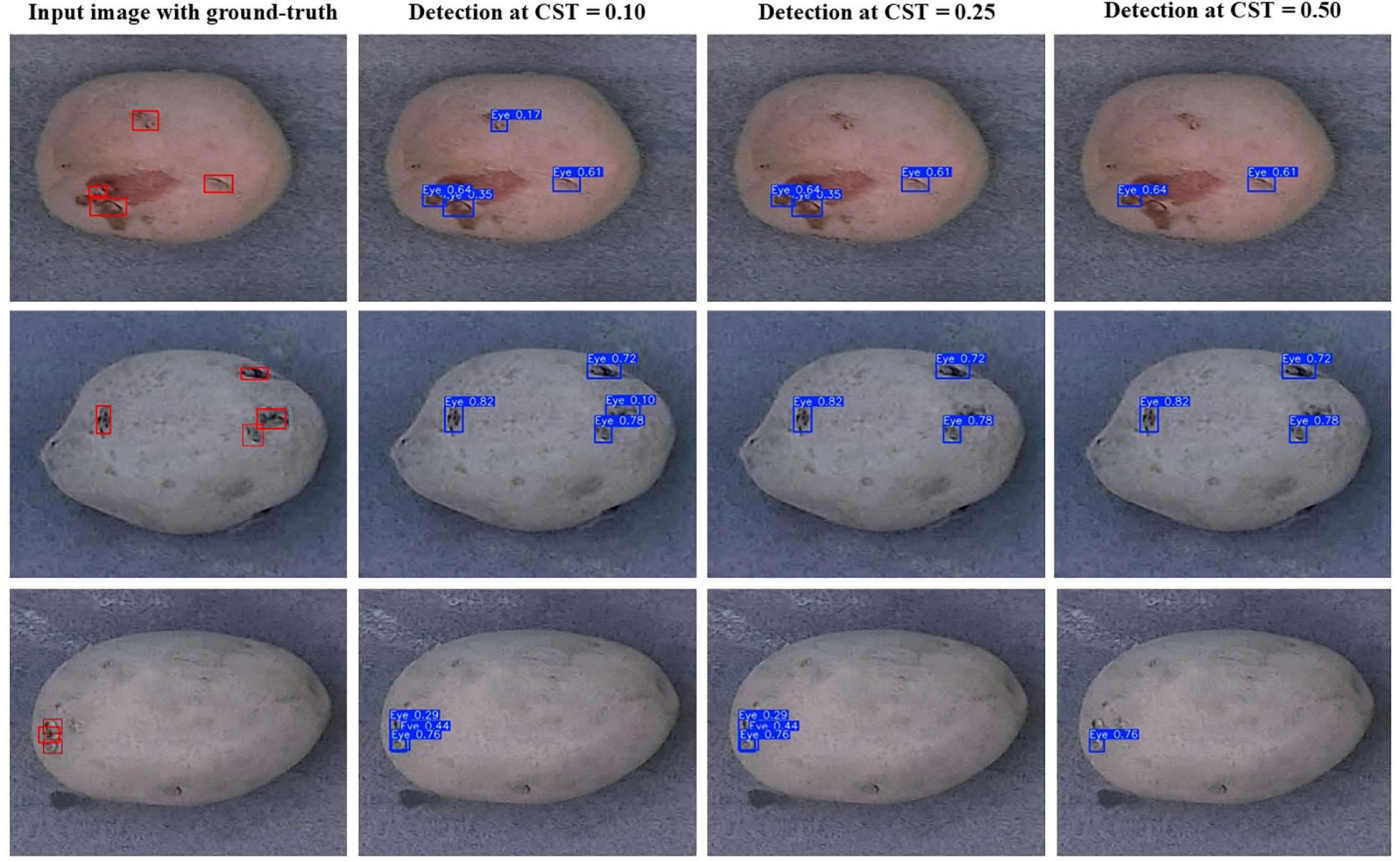
Eye detection results of the YOLOv10m model across different confidence score thresholds (CST) on sample tuber images obtained from tubers of ‘Ciklamen’ (first row), ‘Purple Pelisse’ (second row), and russet cultivars (third row).

As discussed earlier, the tuber sampling operation necessitates minimal false positive errors from the object detection model. The sampling process only requires a tuber core from stolon scar and three eyes per tuber distributed across the entire tuber surface. Consequently, increasing the confidence score threshold of the detection model can lead to very high precision, representing the proportion of correctly predicted positive instances among all instances predicted as positive (**Figure 8**). The sampling process will be performed iteratively prioritizing the instances with the highest prediction confidence while accounting for proximity of other detected eyes, ensuring that representative tissue is collected from across the entire tuber surface. The findings of this study offer valuable guidance for optimizing the selection and deployment of YOLO models for developing a robotic tuber sampling device for the potato industry.

## 5 Conclusion

The comprehensive evaluation of nineteen different versions of YOLO architectures for detection of eyes on potato tubers provided valuable insights for the development of a robotic tuber sampling device. Our findings demonstrate that while all models showed appreciable accuracies, YOLOv10l and YOLOv10m models emerged as the top performers in terms of precision, recall, and mAP scores, closely followed by YOLOv7. Particularly noteworthy is the performance of YOLOv10m, which achieved competitive accuracy with significantly fewer parameters and lower inference time compared to YOLOv7, making it a promising candidate for practical deployment. While certain models exhibit robust performance across a range of cultivars, disparities in detection accuracy were observed, particularly in the cultivar with purple skin. This underscores the need for continued refinement and adaptation of detection models to accommodate the variability inherent among different potato cultivars.

## Data Availability

The tuber image datasets used in this study are publicly available at https://github.com/divyanthlg/tissueSamplingRobot/blob/main/potato_image_dataset.zip.
